# Magnesium-permeable *TRPM6* polymorphisms in patients with meningomyelocele

**DOI:** 10.1186/s40064-016-3395-7

**Published:** 2016-10-03

**Authors:** Mehmet Saraç, Ebru Önalan, Ünal Bakal, Tugay Tartar, Mustafa Aydın, Ayşen Orman, Ahmet Tektemur, Erdal Taşkın, Fatih Serhat Erol, Ahmet Kazez

**Affiliations:** 1 Department of Pediatric Surgery, Firat University Medical Faculty, 23119 Elazig, Turkey; 2 Department of Medical Biology, Firat University Medical Faculty, Elazig, Turkey; 3 Department of Neonatology, Firat University Medical Faculty, Elazig, Turkey; 4 Department of Neurosurgery, Firat University Medical Faculty, Elazig, Turkey

**Keywords:** Neural tube defect, Spina bifida cystica, Etiopathogenesis, Genetic, Embryogenesis, Development, Human, Children

## Abstract

**Background:**

To evaluate whether there is an association between single nucleotide polymorphisms in magnesium-permeable *TRPM6* ion channel and development of meningomyelocele (MMC). Therefore, we examined a total of 150 children with MMC, along with age- and gender-matched controls. DNA collected from whole blood was analyzed for the presence of two polymorphisms, rs2274924 (A > G; K1579E; Leu1579Glu) and rs3750425 (G > A; Val1393Ile), in *TRPM6*. Serum Mg^2+^ and calcium levels were also examined.

**Results:**

A statistically significant difference in the distribution of rs2274924 genotypes (p = 0.049) was observed between the groups. Decreases in the AA genotype, and increases in the AG heterozygous genotype were also detected in the study group. The distribution of polymorphisms in the rs3750425 genotype and alleles was not statistically different between groups. Serum Mg^2+^ levels were lower in the GG genotype of rs3750425 compared with the GA and AA genotypes (p = 0.003).

**Conclusions:**

A statistically significant difference in rs3750425 genotypes was observed between the patients with MMC and the controls, which corresponded to lower serum Mg^2+^ concentrations in these patients. Taken together, these results suggest that genetic variations in the Mg^2+^-permeable *TRPM6* ion channel may play a role in the etiopathogenesis of MMC during embryonic development.

## Background

Neural tube defects (NTDs) occur during embryogenic period when the neural tube lefts uncovered to close normally and neural tissue exposed to the intrauterine environment. It affects about 0.1–0.4 % in worldwide, 0.3 % in Turkey (Mandiracioğlu et al. 2004; van Rooij et al. 2003) and 2.6/1000 in East Anatolia region of Turkey in live births (Saraç et al. 2007). Meningomyelocele (MMC) is a nonlethal but severely disabling form of NTD (Shutleff and Graaf 2001). Etiology of MMC remains poorly understood, most likely driven by a combination of genetic and environmental factors (Miao et al. 2016; Au et al. 2010). Genetic factors may contribute up to 70 % of NTD prevalence based on epidemiological evidences to date (Leck 1974; Copp and Greene 2010; Bassuk and Kibar 2009). Maternal health problems, including inadequate nutrition have also been shown to affect neural tube closure through a variety of mechanisms (Copp et al. 2013).

Magnesium (Mg^2+^) is the second most abundant cation in cell and is essential for all stages of life, from the early embryo to adult. Regulation of cellular as well as whole body Mg^2+^ homeostasis is critical for human health. Although the concentration of Mg^2+^ in the extracellular environment can vary significantly, the total intracellular Mg^2+^ concentration is actively maintained within a relatively narrow range (14–20 mM) via tight (Komiya and Runnels 2015; Romani 2011). This responsibility largely achives to Mg^2+^ transporters and ion channels. Transient receptor potential cation channel, subfamily M, member 6 (*TRPM6*; 602014), located on chromosome 9q21.13, plays a central role in the regulation of Mg^2+^ homeostasis via absorption and reabsorption of Mg^2+^ from the small intestines and kidneys, respectively (Song et al. 2009; Hruby et al. 2013; Groenestege et al. 2006; Schlingmann et al. 2005).

Homozygous TRPM6 knockout embryos died by E12.5 and exhibited neural tube closure defects (Walder et al. 2002). The expression pattern of TRPM6 during embryogenesis demonstrated a significant increase at E10, suggesting TRPM6 is also temporally required for early development. Heterozygous TRPM6 knockout mice exhibited mild hypomagnesemia (Walder et al. 2002; Woudenberg-Vrenken et al. 2011). Mutations in TRPM6 occur in the rare autosomal-recessive disease hypomagnesemia with secondary hypocalcemia (HSH) in human (Schlingmann et al. 2002). As indicated in many studies, hypomagnesemia in null mice is sufficient to induce NTDs, as well as developmental disorders of the bones (Walder et al. 2009; Chubanov and Gudermann 2014). Proper Mg^2+^ levels are also of particular importance to pregnant women, with deficiencies associated with premature birth and intrauterine growth retardation, along with increases in both mortality and morbidity during the embryonic period. Embryotoxic and teratogenic effects of Mg^2+^ have also been reported in animal models. Lower serum Mg^2+^ levels are strongly associated with skeletal malformations, osteogenesis imperfecta, and growth retardation of the embryo, as well as dysregulation of fetal temperature and sudden infant death (Hurley et al. 1976; Günther et al. 1981).

The fundamental principles underlying *TRPM6* ion channel operation have been described in detail. *TRPM7*, an ion channel permeable to both Ca^+^ and Mg^2+^, functions via the formation of heterodimers with TRPM6 (Günther et al. 1981). Single nucleotide polymorphisms (SNPs) of *TRPM6* have been associated with diabetes mellitus and serum magnesium level in human (Song et al. 2009; Hruby et al. 2013; Walder et al. 2009). Given these associations, we sought to investigate the role of rs2274924 (A > G; K1579E;Leu1579Glu) ve Rs3750425 (G > A Val1393Ile) polymorphisms in *TRPM6* comparing with the frequency of specific genotypes and alleles in patients with MMC and healthy controls and the potential correlation between these SNP variants and serum Mg^2+^ and calcium concentrations to identify potential functional outcomes associated with disease genotypes.

## Methods

This case–control study was conducted after obtaining approval from the institutional ethics committee (protocol number 31.01.2013/02/12). Oral and written consent were obtained from all families prior to inclusion in this study. A total of 150 patients diagnosed with MMC admitted to the outpatient clinics of neurosurgery, pediatric surgery, and pediatrics were enrolled in this study. An additional 150 patients admitted to the pediatric surgery outpatient clinic with no evidence of abnormal sacral spine examination findings were used as a control group. The demographic characteristics and clinical history of each patient were investigated in detail. Evidence of consanguineous marriage between parents or earlier generations was also noted. Due to the possibility of teratogenic effects, maternal use of any drug during the periconceptional period was recorded. Serum calcium and Mg^2+^ levels were assessed using a colorimetric assay.

### Isolation of DNA

DNA isolation was performed using the Promega Wizard^®^ Genomic DNA Isolation Kit according to the manufacturer’s instructions. The DNA concentration was measured using a MaestroNano^®^ (MaestroNanodrop; Maestrogen, USA), and diluted to 1–10 ng.

### Genotyping using TaqMan^®^ probes

SNPs of rs2274924 (A > G; K1579E;Leu1579Glu) and rs3750425 (G > A; Val1393Ile) in *TRPM6* were analyzed using TaqMan^*®*^ probes in combination with an ABI 7500 Fast Real-Time PCR System (Applied Biosystems, Foster City, CA, USA). The genomic sequence of rs2274924 was assessed using the probe AGTCCTTGAGTATTCTTCTTTTTCT[C/T]TGACAGTCTCCTGTCTTTGGTTAGC (Assay ID: C_16183233_20). Polymorphism at this nucleotide confers an AAG to GAG substitution, resulting in a leucine to glutamic acid substitution at residue 1579. For genotyping, the kit used FAM and VIC florescent dyes for the A and G alleles, respectively, as forward and reverse primers. The genomic sequence of rs3750425 was assessed using the probe TCCACTGATGCCCAGTCAGAGACAA[C/T]TGGGGTCTGCCCAGTCAGATGAACA (Assay ID: C_25943493_20). Polymorphism at this nucleotide confers an ATT to GAG substitution, resulting in a valine to isoleucine substitution at residue 1393. For genotyping, the kit used FAM and VIC florescent dyes for the A and G alleles, respectively, as forward and reverse primers. All polymerase chain reaction (PCR) reaction mixtures were prepared on ice.

From each sample, we combined 2.5 µL DNA with 7.5 µL PCR reaction mixture for a final volume of 10 µL. Plates were then covered with optical film, centrifuged, and analyzed using a 7500 Fast Real-Time PCR System for 40 cycles. The device software was used to identify homozygous mutant, heterozygous, and homozygous normal genotypes for each allele.

### Statistical analysis

Data were analyzed using the IBM Statistical Package for the Social Sciences v. 21 (SPSS Inc., Chicago, IL, USA). Relationships between parameters were assessed using Pearson’s correlation analysis. Distributions of genotype and allele frequency were analyzed using a Chi square test. The Mann–Whitney *U* test was used to evaluate differences between groups. Data were expressed as the mean ± standard deviation. A *p* value <0.05 was considered statistically significant.

## Results

The study group (n = 150) consisted of 60 female (40 %) and 90 (60 %) male patients. The mean age of the study group was 3.1 ± 3.22 years (range 1 month–18 years). The mean age of the mothers was 28.15 ± 4.71 years (range 19–44 years). A detailed list of demographic, clinical, and laboratory findings are presented in Table 1.

**Table 1:** Demographic, clinical, and laboratory findings of each group

	Study group	Control group
Age (years)	3.1 ± 3.22	9.7 ± 2.21
Female/male	60/90	75/75
History of another baby with MMC in the family	18 (11.9 %)	–
Consanguinity between parents	41 (27.3 %)	15 (10 %)
History of another baby with a congenital anomaly in the family	32 (21.3 %)	10 (6.6 %)
Drug use during pregnancy	25 (16.6 %)	15 (10 %)
Calcium (mg/dL)	9.09 ± 1.24	10.0 ± 1.25
Mg^2+^ (mg/dL)	1.98 ± 0.62	2.3 ± 0.39

*MMC* Meningomyelocele

 No significant differences in genotype or allele distributions of *TRPM6* rs3750425 polymorphisms were evident between the groups (Table 2). In contrast, a statistically significant difference was observed between the study and control groups in terms of the distribution of the rs2274924 genotype (p = 0.049). Prevalence of the AA genotype was markedly decreased in patients with MMC, while the heterozygous AG genotype was increased. However, no significant difference in the distribution of rs2274924 alleles was observed between the groups (Table 2).

**Table 2:** Frequencies of the genotypes and alleles of rs3750425 and rs2274924 polymorphisms in the transient receptor potential cation channel, subfamily M, member 6 (*TRPM6*) gene

	Genotypes			*p* value	Odds ratio (95 %)
	GG	GA	AA		
rs3750425 Polymorphism groups					
Study group	94	53	3	0.14	1.45 (0.88–2.39)
Control group	103	40	7		

	Alleles				
	G	A			
Study group	241	59	0.68	1.11 (0.74–1.67)	
Control group	246	54			

	Genotypes				
	AA	AG	GG		
rs2274924 Polymorphism groups					
Study group	71	66	13	0.049	1.84 (1.13–3)
Control group	91	46	13		

	Alleles				
	A	G			
Study group	214	86	0.22	1.27 (0.88–1.83)	
Control group	228	72			

*GG* wild type, *GA* heterozygous, *AA* polymorphic allele, *AA* wild type, *AG* heterozygous, *GG* polymorphic allele

Within the study group, we observed a statistically significant difference in serum Mg^2+^ levels associated with rs3750425 genotypes but not for rs2274924 genotype. Serum Mg^2+^ levels were significantly lower in patients harboring the rs3750425 GG genotype relative to those possessing either the GA or AA genotypes (p = 0.003; Table 3). No differences were observed in relation to rs2274924 genotypes.

**Table 3:** Mean serum Mg^2+^ and Ca^+^ levels in patients with meningomyelocele (MMC) according to their genotype

Polymorphism	Calcium		*p* value	Mg^2+^	*p* value
rs3750425	GG	9.2 ± 1.20	0.48	1.51 ± 0.31	0.003
	GA	9.0 ± 1.21		2.0 ± 0.34	
	AA	9.5 ± 1.18		2.1 ± 0.38	
rs2274924	AA	9.35 ± 1.17	0.33	1.80 ± 0.41	0.37
	AG	9.02 ± 1.15		1.82 ± 0.48	
	GG	9.30 ± 1.2		1.81 ± 0.42	

## Discussion

Our statistical finding focused on two coding SNPs in *TRPM6* in NTD patients. TRPM6 polymorphism may contribute to different ways the NTD development, such as regulating osteogenesis during embryonic development, effecting the maternal serum magnesium level in pregnant women and changing in the expression level under the influence of estrogen hormone during pregnancy.

A limited number of studies have investigated the potential links between *TRPM6* variations and human disease. In our study population, approximately 31.5 % are heterozygous for the rare *G* allele of rs2274924 and 22 % heterozygous for the allele *A* of rs3750425. The HapMap studies were shown that population frequencies are 0.07–0.21 for the rs3750425 *A* allele (1393 Ile) and 0.15–0.36 for the rs2274924 *G* allele (1584Glu) in four ethnic groups (Walder et al. 2009). Song et al. (2009) provide suggestive evidence that two common non-synonymous TRPM6 coding region variants, Ile1393Val (rs3750425) and Lys1584Glu (rs2274924) polymorphisms, might confer susceptibility to type 2 diabetes in women with low magnesium intake. However, a follow-up study of Romero et al. (2010), Shuen et al. (2009) failed to duplicate these results for rs3750425 and rs2274924. A significant association was observed between common variants of *TRPM6* and hypomagnesemia (Hruby et al. 2013; Meyer et al. 2010), with a specific T allele of rs11144134 in *TRPM6* associated with higher femoral neck and lumbar spine BMD (Meyer et al. 2010). Magnesium-deficiency has been linked to osteoporosis and low BMD in observational and animal studies (Alexander et al. 2008). Disorders in osteogenesis responsible from inhibition of bone matrix mineralization (Semczuk and Semczuk-Sikora 2001; Durlach 2004; Takaya et al. 2006). The transcripts of canonical TRPM6 channel were also revealed in human MG-63, SaOS and U2 OS osteoblast cell lines (Abed et al. 2009). These observations show that TRPM6 may important the control of bone homeostasis during embryonic and adulthood. Polymorphic amino acids for two polymorphisms investigated in present study are located between the coiled region and kinase near the C-terminal (Schlingmann et al. 2004). Although these amino acid changes in coiled region of TRPM6 protein are improbable responsible for direct regulation of trans-membrane structures and kinase activity, they may alter protein conformation and thus decrease TRPM6 channel activity.

Mg^2+^ deficiency causes or contributes to some abnormalities during embrionic development in human and rats. The decreased maternal Mg^2+^ intake because of inadequate nutrition and embryonic genetic defects might be responsible from Mg^2+^ deficiency. A maternal Mg^2+^ intake below 378 mg/day has been associated with a twofold to threefold higher risk for the neural tube defect, spina bifida in offspring, suggesting a pivotal role for Mg^2+^ not just in growth but in morphogenesis as well (Groenen et al. 2004). A decrease in basal [Mg^2+^]i from cord blood platelets is associated with small for gestational age (SGA) babies in humans (Venu et al. 2008). Oral Mg^2+^ supplementation given before the 25th week of gestation is also associated with a lower frequency of preterm births, a lower frequency of low birth weight, and fewer SGA infants compared with placebo (Makrides and Crowther 2001). Pups of pregnant females fed a Mg^2+^ deficient diet demonstrated an increased incidence of resorptions, gross malformation, growth retardation, abnormal fat metabolism, insulin resistance and diabetes (Hurley et al. 1976; Venu et al. 2008). Despite these apparent links between maternal serum Mg^2+^ levels and some embryonic and fetal anomalies, no correlation was found between maternal or neonatal serum Mg^2+^ levels and MMC (Golalipour and Mansourian 2010). These observations are broadly consistent with the results presented here, with no significantly different neonatal serum Mg^2+^ or Ca^+^ levels evident between the groups, although lower serum Mg^2+^ levels were detected in patients with GG genotypes in rs3750425 compared with either the GA or AA variants. As Mg^2+^ homeostasis in the embryo is critically dependent on the maternal blood supply, a lack of genetic and biochemical analysis of maternal serum samples to assess whether Mg^2+^ homeostasis was altered during prenatal development was the main limitation of our study.

Recent studies have continued to add to the growing number of Mg^2+^ transporters and ion channels involved in Mg^2+^ homeostasis during embryogenesis. The expression pattern of TRPM6 during embryogenesis demonstrated a significant increase at E10, suggesting TRPM6 is also temporally required for early development. Heterozygous TRPM6 knockout mice exhibited mild hypomagnesemia (Walder et al. 2002; Woudenberg-Vrenken et al. 2011). Interestingly, in one of the studies dams fed with a high Mg^2+^ diet slightly suppressed the embryonic lethality caused by knockout of TRPM6, suggesting that embryonic lethality may in part be due to Mg^2+^ deficiency. However, in the second study by Woudenberg-Vreken et al. (2011) a high Mg^2+^ diet failed to rescue the lethality caused by homozygous deletion of *TRPM6*. Rare mutations or polymorphic variants in TRPM6, including amino acid substitutions that prevent its heterooligomerization with TRPM7 or changing channel activity, may cause the imbalance in intracellular magnesium level and likely to contribute the abnormal bone development during embryogenesis.

Recent studies have shown that estrogen directly increases the transcription of *TRPM6*, as well as its activity (Cao et al. 2009); this effect is of particular importance during pregnancy, due to the marked increase in maternal estrogen levels. As 90 % of maternal estriol consists of fetal precursors, these effects take on added significance (Buster et al. 1976). Given the strong evidence that estrogen influences *TRPM6* synthesis during pregnancy, this gene is likely to play an important role in the regulation of Mg^2+^ homeostasis in both the mother and fetus. Genetic variations in *TRPM6* may therefore affect intracellular Mg^2+^ levels during embryonic development, with significant implications for fetal development.

Several limitations in present study must be completed with further consideration. First, our study had relatively small sample size, however, consistency with laboratory findings makes our results acceptable. Second, population’s etnic diversity may be a concern but imposible to clarify our findings, because our populations are racially homogeneous, all participant lived in Elazig region. Third, althought serum magnesium may be a good indicator for heavy magnesium deficiency, we didn’t performed the measures of extracellular or intracellular magnesium status and the patch clamp experiment to display the effect on channel activity of studying polymorphic variant in TRPM6 gene.

Clinical data show that sufficient magnesium intake can partially compensate for the severe magnesium deficiency caused by genetic defect in the *TRPM6* gene (Altıncık et al. 2016). High magnesium concentrations in intestinal tracts may overcome the genetic defects of magnesium absorption and independently increase magnesium absorption via the other pathways. Inadequating of dietary magnesium intake, the function of *TRPM6* in active intestinal magnesium absorption and renal reabsorption happen very important. Based on these observations, we strongly recommend checking serum Mg^2+^ values of babies born with MMC. Further studies performed on a large number of patients will be necessary to confirm these findings, as well as clarify the etiology of MMC, particularly in the context of Mg^2+^ homeostasis.
